# NF-κB as an Important Factor in Optimizing Poxvirus-Based Vaccines against Viral Infections

**DOI:** 10.3390/pathogens9121001

**Published:** 2020-11-29

**Authors:** Justyna Struzik, Lidia Szulc-Dąbrowska

**Affiliations:** Division of Immunology, Department of Preclinical Sciences, Institute of Veterinary Medicine, Warsaw University of Life Sciences-SGGW, Ciszewskiego 8, 02-786 Warsaw, Poland; lidia_szulc@sggw.edu.pl

**Keywords:** poxvirus, vaccine vector, viral infection, NF-κB

## Abstract

Poxviruses are large dsDNA viruses that are regarded as good candidates for vaccine vectors. Because the members of the *Poxviridae* family encode numerous immunomodulatory proteins in their genomes, it is necessary to carry out certain modifications in poxviral candidates for vaccine vectors to improve the vaccine. Currently, several poxvirus-based vaccines targeted at viral infections are under development. One of the important aspects of the influence of poxviruses on the immune system is that they encode a large array of inhibitors of the nuclear factor kappa-light-chain-enhancer of activated B cells (NF-κB), which is the key element of both innate and adaptive immunity. Importantly, the NF-κB transcription factor induces the mechanisms associated with adaptive immunological memory involving the activation of effector and memory T cells upon vaccination. Since poxviruses encode various NF-κB inhibitor proteins, before the use of poxviral vaccine vectors, modifications that influence NF-κB activation and consequently affect the immunogenicity of the vaccine should be carried out. This review focuses on NF-κB as an essential factor in the optimization of poxviral vaccines against viral infections.

## 1. Introduction

*Poxviridae* is a family of dsDNA viruses. It is divided into two subfamilies: *Chordopoxvirinae*, the viruses of vertebrates, and *Entomopoxvirinae*, the viruses of insects. The *Chordopoxvirinae* subfamily includes 18 genera: *Avipoxvirus*, *Capripoxvirus*, *Centapoxvirus*, *Cervidopoxvirus*, *Crocodylidopoxvirus*, *Leporipoxvirus*, *Macropopoxvirus*, *Molluscipoxvirus*, *Mustelpoxvirus*, *Orthopoxvirus*, *Oryzopoxvirus*, *Parapoxvirus*, *Pteropopoxvirus*, *Salmonpoxvirus*, *Sciuripoxvirus*, *Suipoxvirus*, *Vespertilionpoxvirus*, and *Yatapoxvirus* [1]. Poxviruses are represented by numerous human and animal pathogens. Among them, variola virus (VARV) orthopoxvirus, a human pathogen, is the causative agent of smallpox, a disease that had caused over 300 million deaths worldwide by the late 1970s before the global smallpox eradication program was completed. In the global smallpox eradication program, vaccinia virus (VACV), a zoonotic pathogen belonging to the *Orthopoxvirus* genus, was used [2,3]. Other members of the *Poxviridae* family, such as orf virus (ORFV) and goatpoxvirus (GTPV), which represent the *Parapoxvirus* and *Capripoxvirus* genera, respectively, may also serve as vaccines and are described in this review.

With the exception of parapoxviruses, poxvirus virions have a brick shape. The virions of parapoxviruses are cocoon-shaped. The virions of parapoxvirus and other members of the *Poxviridae* family have dimensions of 260 × 160 nm and 350 × 250 nm, respectively [4,5]. Depending on the number of membranes surrounding the virion, two infectious forms of poxviruses are observed. Mature virus (MV), which contains a tubular nucleocapsid surrounded by a biconcave core wall and proteinaceous lateral bodies, is enclosed by a single proteolipid membrane bilayer. In turn, extracellular virus (EV) is composed of MV surrounded by an additional membrane derived from an early endosome or the trans-Golgi. This membrane is acquired by the virus during exocytosis [6].

The genome of poxviruses ranges from 130 to 300 kbp. The largest genome can be observed among avipoxviruses, whereas the smallest can be observed in parapoxviruses [4,5]. The genes encoding the open reading frames (ORFs) linked to virus replication, such as those essential for the nucleic acid synthesis and structural components of the virion, are located within the conserved central region of the poxvirus genome. These genes encode DNA polymerase, DNA ligase, DNA-dependent RNA polymerase, as well as the enzymes involved in capping and polyadenylation of mRNAs, and thymidine kinase (TK). The genes flanking the central region of the poxvirus genome encode numerous proteins that determine the host range and virulence and are responsible for modulating the immune response of the host. The two DNA strands of poxvirus genome are joined together by covalent linkage at both ends, where inverted terminal repetitions (ITRs), which are long tandem repeated nucleotide sequences flanking the genome, are present [5].

The ORFs present at the terminal poxviral genome mainly target the innate immune response mechanisms of the host via modulation of the antiviral signaling pathways. One of the innate antiviral pathways modulated by poxviruses is the stimulator of interferon (IFN) genes (STING) pathway that senses the viral dsDNA. Cytosolic sensors of viral DNA, namely cyclic GMP–AMP synthase (cGAS), DNA-dependent protein kinase (DNA-PK), and IFN-γ-inducible protein 16 (IFI16), activate the STING adaptor protein, and this protein, in turn, activates tumor necrosis factor (TNF) receptor (TNFR)-associated factor (TRAF) nuclear factor kappa-light-chain-enhancer of activated B cells (NF-κB) (TANK)-binding kinase 1 (TBK1)–IFN regulatory factor 3 (IRF3) and inhibitor κB (IκB) kinase (IKK)–NF-κB pathways which are crucial for antiviral response. These pathways induce the synthesis of immune defense molecules, such as proinflammatory cytokines and IFNs. It has been demonstrated that VACV encodes C4 and C16 proteins which may antagonize DNA–PK and thus impair cytokine response and IRF3 inhibition. C16, which acts upstream of STING, inhibits its activation. In addition, B2 VACV has been found to target cGMP. C4 may also inhibit NF-κB activation. Importantly, the inhibition of NF-κB by poxviruses may be multidirectional and occur downstream of STING activation [7,8].

Deletion of B2 or the antagonist of VACV DNA-PK could be beneficial for vaccine development. Thus, studies on DNA sensing pathways may shed light on the potential therapeutic strategies. Knowing that certain VACV proteins, such as K1 and A55, prevent the nuclear translocation of NF-κB or NF-κB heterodimer processing, it should be considered that they may also inhibit STING-induced NF-κB activation [7]. Taken together, studying the modification of NF-κB and other related cellular signaling pathways by VACV and other poxviruses may help in finding novel options for the modification of vaccine vectors.

## 2. Poxviruses as Candidates for Vaccine Vector Design

Poxviruses are transmitted via mucosal, respiratory, and parenteral routes [9]. Although these viruses may enter various cell types, only the cells supporting their full replication cycle can be considered as permissive for the infection. The genomes of many poxviruses share similar sequences; however, during their evolution, the loss or truncation of certain genes, which confer the full replication cycle of the poxviruses, influenced their host range [9,10,11].

Poxviruses serve as good vaccine vectors due to the fact that a large sequence of up to 25 kbp encoding viral, bacterial, parasitic, and tumor antigens can be introduced into their genomes. These heterologous antigens are aimed at triggering antibody response and inducing cytotoxic T lymphocytes (CTLs) to confer immunity. As mentioned earlier, poxviral vectors can infect different types of cells [12,13,14]. The lifecycle of poxviruses takes place in the cell cytoplasm, within the cytoplasmic compartments called viral factories. Poxviral pathogens encode factors needed for DNA replication, transcription, mRNA processing, and cytoplasmic redox systems. However, like other viruses, poxviruses are fully dependent on host ribosomes, which are required for mRNA translation [15,16,17,18,19,20,21,22,23,24,25].

One of the advantages that make poxviruses good vaccine vectors is that the cytoplasmic replication cycle of these vectors eliminates the risk of integration into the host genome and persistence within the host. Importantly, poxviral vaccines are easy to store, especially when freeze-dried. The thermostability of these vaccines can also be ensured by using sugar-glass technology. Additionally, the cost of poxviral vaccines is low and their administration is needle-free [12,13,14]. 

Although poxviruses are regarded as promising vaccine tools, certain challenges limit the design of poxviral vaccines. When using VACV and other poxvirus-based vaccines, it is desirable to achieve enhanced immunogenicity and/or virus attenuation. This is particularly important for improving the safety profile of the vaccine. Due to the abundance of immunomodulatory genes and cellular targets of the poxviruses, which remain unrevealed, there are still many opportunities for virus modification in order to improve the vaccine efficacy by inducing stronger immunological memory. In addition, reduction of dosage and administration regimes would be beneficial as well [7]. One of the strategies employing poxvirus vaccines is prime-boost vaccination, in which poxviral vectors that enhance T cell responses as boosters are combined with other vectors. On the other hand, when used as primers with protein and adjuvant, poxviruses improve the B cell responses. Furthermore, the optimization of the antigen expression is based on mosaic immunogen sequences [13]. 

When modifying poxviral vaccine vectors, the immunomodulatory genes should be removed in order to enhance immunogenicity [13]. Poxviruses, which express a wide range of host response modifiers influencing cellular signaling pathways involved in immunity and inflammation, share multiple mechanisms of host evasion. Since *Poxviridae* family members encode a number of cellular signaling inhibitors, this review describes the influence of poxvirus-based vaccines on the NF-κB transcription factor [9]. Several data indicate the importance of NF-κB in the development of poxvirus-derived vaccines. These are based on both veterinary and human antiviral vaccines. Therefore, in this review, we focus on the benefits of certain modifications of poxviral vaccine vectors and how these modifications can affect NF-κB signaling in different cells and hosts and the possible mechanisms of immune response modulation that can be shared by individual poxvirus genera. We describe the VACV-, ORFV-, and GTPV-based vaccines, which can be used against viral infections.

## 3. Poxviral Vectors for Vaccine Applications

The vaccine used in the global smallpox eradication program was based on several strains of VACV. For instance, in the United States, the New York City Board of Health (known as NYCBH) and Lister strains were used for vaccination against smallpox, while in Europe, Lister, Bern, Paris, and Copenhagen (called VACV-COP) strains were applied. The first-generation antismallpox vaccines were propagated in the skin of calf and other animals, while the second-generation VACV-based vaccines were grown in tissue culture and chicken embryos instead of live animals. Unfortunately, the vaccines generated in cell cultures are not sufficiently safe. Therefore, the use of second-generation antismallpox vaccines is limited [3]. Recently, it has been shown that chicken embryonic stem cells (cESCs) may serve as an alternative source for the propagation of poxvirus vaccine vectors. The idea behind the use of cESCs rather than mammalian ESCs is linked to ethical issues and safety concerns, the most important of which is that cESCs lack transforming oncogenes or adventitious agents [26].

Despite the eradication of smallpox, the risk of reoccurring VARV infections remains to be eliminated because of the bioterrorist threat or the possible de novo synthesis of the virus. Therefore, the pathogenesis of orthopoxvirus diseases is still of interest to researchers [27,28]. Although VACV-based vaccines used in the global smallpox eradication program were effective, some adverse effects of vaccination, including postvaccinal encephalitis, generalized vaccinia, progressive vaccinia, and eczema vaccination, were observed in immunocompromised individuals and patients with skin conditions. To overcome these, modified VACV Ankara (MVA)—Bavarian Nordic (MVA-BN), a third-generation attenuated antismallpox vaccine—has been introduced. This vaccine is approved in Canada (Imvamune) and the European Union (Imvanex) [29,30]. 

An important concern related to smallpox is not only the fear of its reoccurrence but also the occurrence of new zoonotic orthopoxviral infections and the disappearance of antismallpox immunity, which confers cross-protection against other orthopoxviral diseases. Zoonotic poxviral infections are caused by VACV, monkeypox virus (MPXV), cowpox virus (CPXV), camelpox virus (CMLV) orthopoxviruses, bovine papular stomatitis virus (BPSV), ORFV, pseudocowpox virus (PCPV) parapoxviruses, Yaba monkey tumor virus (YMTV), and tanapox virus (TPV) yatapoxviruses. Therefore, antiviral drugs and vaccines against poxviral diseases are still under development [31,32,33,34,35,36,37,38,39,40].

The currently used VACV-based vaccines, which are nonreplicating and attenuated, such as MVA-BN-vectored encephalitic alphavirus vaccine, target the biothreat viruses. Another VACV-derived vaccine, RABORAL V-RG, an antirabies vaccine expressing the rabies virus (RABV) glycoprotein gene (V-RG), has been used in Europe and North America to vaccinate foxes and raccoons [30]. Additionally, VACV-based vaccine candidates have been demonstrated to protect against emerging viral diseases, such as chikungunya virus (CHIKV) disease [41,42] and yellow fever [43] in preclinical animal models. The Sementis Copenhagen Vector (SCV) is a new multiplication-defective VACV-COP-derived vaccine vector with targeted deletion of *D13L* gene encoding D13 protein that is essential for viral assembly. This new vector has recently been successfully tested in nonhuman primates as a vaccine against Zika and chikungunya [41].

The present status of clinical trials employing VACV against viral diseases is shown in Table 1. Other poxviruses that can be used as vaccine vectors belong to *Avipoxvirus* [44,45], *Capripoxvirus* [46], *Leporipoxvirus* [47], *Parapoxvirus* [48], and *Suipoxvirus* [49] genera.

## 4. NF-κB Signaling

**Figure 1:**
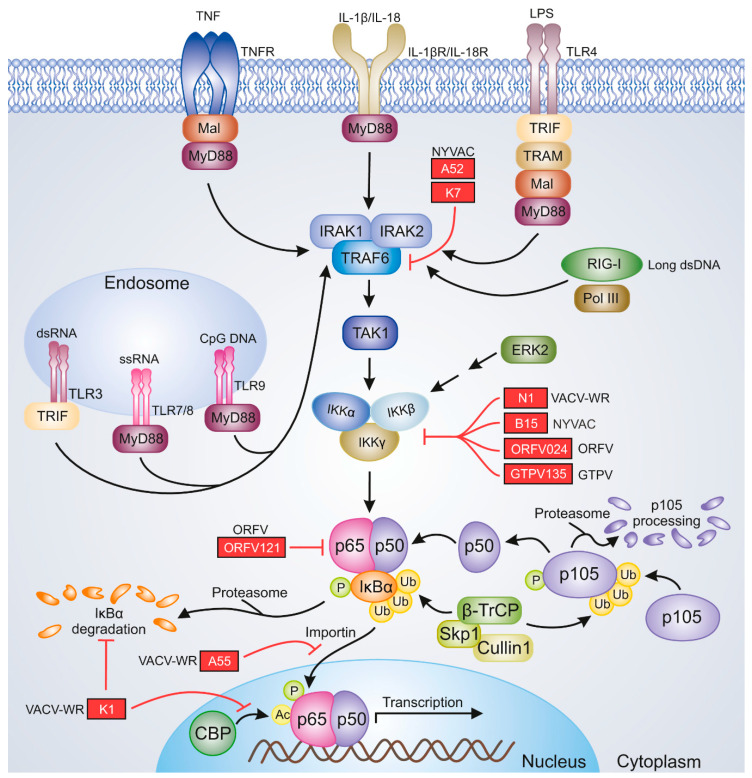
Poxviral inhibitors of NF-κB signaling. The image represents selected viral proteins that block NF-κB activation. The proteins shown in the figure are described in the text. Black pointing arrows indicate activation; red blunt arrows indicate inhibition. Ac, acetyl group; CBP, CREB-binding protein; CpG, cytosine–guanine dinucleotide; ERK2, extracellular signal-regulated kinase 2; GTPV, goatpox virus; IKKα, IκB kinase α; IKKβ, IκB kinase β; IKKγ, IκB kinase γ; IL-1β, interleukin 1β; IL-18, interleukin 18; IL-18R, IL-18 receptor; IL-1βR, IL-1β receptor; IRAK1, IL-1R-associated kinase 1; IRAK2, IL-1R-associated kinase 2; IκBα, inhibitor κBα; LPS, lipopolysaccharide; Mal, MyD88-adapter-like; MyD88, myeloid differentiation primary response gene 88; NYVAC, vaccinia virus New York strain; ORFV, orf virus; P, phosphate group; Pol III, polymerase III; RIG-I, retinoic acid-inducible gene; Skp1, S-phase kinase-associated protein 1; TAK1, transforming growth factor (TGF)β-activated kinase 1; TLR3, Toll-like receptor 3; TLR4, Toll-like receptor 4; TLR7, Toll-like receptor 7; TLR8, Toll-like receptor 8; TLR9, Toll-like receptor 9; TNF, tumor necrosis factor; TNFR, TNF receptor; TRAF6, TNFR-associated factor 6; TRAM, TRIF-related adapter molecule; TRIF, Toll-IL-1R-domain-containing adapter-inducing interferon-β; Ub, Ub-ubiquitin moieties; VACV-WR, vaccinia virus Western Reserve strain; β-TrCP, β-transducin repeat-containing protein.

One of the key factors involved in the proper induction of antiviral immunity is NF-κB. It constitutes a family of dimeric transcription factors, which regulate the expression of numerous genes involved in the cell cycle, apoptosis, and immunity. The NF-κB family consists of five proteins: RelA/p65, RelB, c-Rel, NFκB1 p105/p50, and NFκB2 p100/p52. The NF-κB dimer that is most commonly detected in the cytoplasm of unstimulated cells is composed of RelA and p50 subunits [50]. The RelA/p50 heterodimer remains in the cytoplasm due to the activity of IκBα, which masks the nuclear localization sequences (NLSs) of NF-κB [51]. The classical NF-κB signaling pathway is induced by proinflammatory cytokines such as interleukin-1β (IL-1β), IL-18, and TNF-α and various ligands of pattern recognition receptors (PRRs), which are represented by retinoic acid-inducible gene-I (RIG-I) and Toll-like receptors (TLRs). In the NF-κB signaling cascade, the cellular receptors cooperate with adapter molecules and induce the cellular pathways that activate the transcriptionally active dimers [52]. Upon the stimulation of NF-κB signaling, transforming growth factor (TGF)-β-activated kinase 1 (ΤAΚ1) activates IKK, the IKKβ subunit of which triggers IκBα phosphorylation at Ser32 and Ser36. This event results in the recognition of IκBα by the E3 ubiquitin ligase complex composed of β-transducin repeat-containing proteins: S-phase kinase-associated protein 1 (Skp1)–Cullin 1–F-box (SCF^β−TrCP^). Conjugation of phosphorylated IκBα with K48-linked polyubiquitin chains of Lys 48 of ubiquitin by SCF^β−TrCP^ results in 26S proteasome-mediated IκBα degradation and the release of RelA/p50 dimers. These dimers translocate to the nucleus, where they bind DNA and initiate the transcription of target genes. E3 ubiquitin ligase complex is also involved in p105 proteasomal processing to p50 [50,53,54] (Figure 1). On the other hand, the noncanonical NF-κB signaling triggered by the members of the TNF superfamily leads to the activation of NF-κB-inducing kinase (NIK), which then activates IKKα. IKKα, in turn, phosphorylates the C-terminal portion of p100 precursor protein, which retains RelB in the cytoplasm due to its IκB activity. Following the phosphorylation of p100 at Ser866 and Ser870, IκB-like C-terminal portions of this protein are ubiquitinated, leading to the generation of a p52 active NF-κB subunit. RelB/p52 dimers translocate to the nucleus and initiate the transcription of target genes [55]. In general, the canonical NF-κB signaling is responsible for the regulation of innate immunity [56], whereas the noncanonical NF-κB activation pathway regulates the adaptive immune responses. However, there exist regulatory mechanisms for these two signaling pathways as well as for the crosstalk between them [55,57]. The modulation of NF-κB signaling is attributed to viral pathogens, one excellent example of which is the viruses belonging to the *Poxviridae* family encoding multiple immunomodulatory proteins; these proteins affect the components of NF-κB signaling and therefore disrupt the antiviral innate response [52,58]. Selected NF-κB inhibitors of VACV, ORFV, and GTPV, which may be relevant to the efficacy of poxviral vaccines, are shown in Figure 1.

## 5. NF-κB in Optimization of Poxviral Vaccines

### 5.1. Vaccinia Virus

VACV is a pathogen whose origin or natural host has not been identified so far. It was believed that VACV infections occur due to the spread of vaccine strains into new wild hosts. However, it is now obvious that VACV is transmitted via peridomestic rodents, which infect wild animals and cows. In humans, VACV can be transmitted via infected animals and is observed in milkers in areas where the virus circulates, especially in Asian and South American countries, such as Brazil. Furthermore, VACV infection of humans occurs via their direct contact with the crusts containing viral particles, which results in the formation of skin focal lesions on the hands and forearms [18,37,59]. After a few days, the focal lesions form pustules leading to edema and erythema. Ulcerated and necrotic lesions appear after a maximum of 12 days of VACV infection, following which crusts develop. In 4 weeks, the lesions disappear, but local lymphadenopathy can be observed for 20 days. Alternatively, a systemic infection manifested by fever, headache, and muscle ache develops immediately after the appearance of lesions [60].

#### 5.1.1. Modified VACV Ankara

VACV is regarded as a universal vaccine carrier. However, the use of replicating VACV as an antismallpox vaccine has led to severe adverse effects in individuals with an immunocompromised immune system or skin disorders. Currently, MVA obtained by 570 passages of chorioallantois VACV Ankara (CVA) strain in chicken embryo fibroblasts remains an excellent alternative to traditional antismallpox vaccines [61]. MVA does not replicate in human cells but displays good immunogenicity as well as a good safety profile in vivo. In addition, MVA can be successfully used in immunocompromised humans [12,15,32,62,63,64,65,66]. The ongoing clinical trials on MVA-vectored vaccines targeted at viral diseases are shown in Table 1. 

MVA is a good candidate for an effective and safe vaccine vector. Nevertheless, its immunogenicity can be enhanced to improve the efficacy of the vaccine, for which the introduction of immune-stimulating genes into MVA and reinsertion of certain VACV genes to obtain a replication-competent virus are considered beneficial [67]. 

Despite the expression of A46 (Toll/IL-1-receptor signaling interference protein), B16 (IL-1-binding protein (IL-1BP)), and Κ7 (B cell lymphoma 2 (Bcl-2)-like protein), which inhibit NF-κB signaling, MVA stimulates NF-κB [61,68,69]. Studies on NF-κB signaling in MVA-infected cells have shown that early MVA protein expression in human 293T fibroblasts activates the phosphorylation of extracellular signal-regulated kinase 2 (ERK2), which, in turn, mediates the activation of NF-κB [70]. Further research revealed that MVA triggers NF-κB activation via VACV growth factor (VGF), which interacts with the epidermal growth factor receptor (EGFR). Moreover, in 293T cells and Hacat keratinocytes infected by MVA deprived of an early *C11R* gene encoding VGF, reduced activation of ERK2 and NF-κB was observed. Since keratinocytes are the immediate target of the vaccine, the prosurvival role of NF-κB would be beneficial to stimulate cells for a long duration for optimal induction of immune response before cell lysis occurs [71].

Other studies on MVA revealed that, during the early phase of viral replication in human embryonic kidney 293 cells that were transformed with large T antigen (HEK 293T), IκBα degradation occurs before the initiation of viral replication. It has been shown that in Chinese hamster ovary (CHO), HEK 293T, and rabbit kidney 13 (RK13) cells, IκBα degradation can be inhibited by the expression of *CP77*, a CPXV Brighton Red (CPXV-BR) early host-range gene [72].

*CP77*-encoded protein is one of the ankyrin (ANK) repeat proteins [73]. In general, ANK repeats, consisting of 30–34 amino acid residues, are involved in protein–protein, protein–sugar, or protein–lipid interactions. ANK repeat proteins take part in cellular signaling, vesicular trafficking, cell cycle control, and inflammation, are responsible for cytoskeleton integrity, and regulate transcription. They are present in both eukaryotic and prokaryotic cells, such as intracellular bacteria [74].

ANK repeat proteins are not commonly expressed by viruses. However, within the *Chordopoxvirinae* subfamily of poxviruses, only three species of different genera lack ANK proteins. In poxviruses, these proteins are composed of multiple ANK motifs, which start from the N-terminus. The ANK motifs are followed by non-ANK linker sequence. In turn, the C-terminus has the homolog of cellular F-box sequence. The cellular F-box sequence interacts with ubiquitin ligase E3 complexes, thus allowing ubiquitination and subsequently 26S proteasome-mediated protein degradation. ANK proteins of poxviruses belonging to *Avipoxvirus*, *Parapoxvirus*, *Orthopoxvirus*, and the *Leporipoxvirus* supergroup genera may either bind to Skp1 or interact with cellular proteins, thus acting as inhibitors of cellular signaling, such as NF-κB [75]. Some poxviral ANK repeat proteins, including VACV K1 [76] and myxoma virus (MYXV) M150 [77], may act as nuclear NF-κB inhibitors. 

Furthermore, certain poxviral ANK repeat proteins, in which the F-box domain is absent, serve as host-range proteins [75]. These are represented by VACV K1 protein encoded by *K1L* gene [78]. It has been shown that MVA expressing the VACV Western Reserve (VACV-WR) gene *K1L*, which prevents IκBα degradation [79] and p65 acetylation [76], potentially inhibits IκBα degradation in HEK 293T cells. Importantly, in mouse embryonic fibroblasts (MEFs), human or mouse dsRNA-activated protein kinase R (PKR) is crucial for IκBα degradation. It can be assumed that the induction of PKR by MVA may stimulate immune response and impair virus replication, which is critical for the safety and efficacy of the vaccine [72].

Furthermore, stimulation of immune response is necessary for the prolonged presentation of antigens and delayed viral clearance. The insertion of a VACV-CVA 5.2-kb region containing apoptosis inhibitor and ANK repeat protein gene *M1L* [80] and NF-κB inhibitors encoded by *M2L* (mitogen-activated protein kinase kinase (MEK)/ERK and NF-κB inhibitor) and *K1L* (PKR and NF-κB inhibitor) genes into MVA decreases both apoptosis and virus-mediated NF-κB activation in antigen-presenting cells (APCs) in vivo. Moreover, VACV-specific CD8^+^ T cell response is diminished in vivo after treatment with MVA/5.2 kb compared to MVA. These results contradict the findings observed in vitro, in which MVA/5.2 kb displays an immunostimulatory effect [67].

#### 5.1.2. New York VACV

Another VACV-based vaccine vector, the New York VACV strain (NYVAC), derived from the VACV-COP strain, is deprived of 18 ORFs, including the host-range *K1L* gene, encoding an IFN antagonist protein and NF-κB inhibitor. It has been shown that both MVA and NYVAC-infected HeLa cells display enhanced expression of NF-κB protein, degradation of IκBα, and secretion of IL-6. Furthermore, NYVAC upregulates certain NF-κB-responsive genes, including activating transcription factor 3 (ATF3). It can be concluded that ATF3 may act pro-apoptotically during NYVAC infection. However, the ectopic expression of VACV-WR *K1L* gene in NYVAC-infected cells induced apoptosis but inhibited NF-κB. This indicates that K1 does not prevent apoptosis in NYVAC-infected cells. Both induction of apoptosis and inhibition of NF-κB may not be favorable for the NYVAC replication cycle; however, apoptosis occurs at the late stage of the viral lifecycle when the replication is completed. These events are important for the generation of immune response and vector clearance [81]. Interestingly, although both MVA and NYVAC stimulate NF-κB, *A52R* gene is present in NYVAC, but not in MVA. The absence of A52, a Bcl-2-like protein, which binds IL-1 receptor (IL-1R)-associated kinase 2 (IRAK2) and thus inhibits NF-κB, is likely to upregulate the TLR3 pathway in MVA-infected cells. Since A52 binds to IRAK2 and TNFR-associated factor 6 (TRAF6), which are important for TLR signaling, upregulation of TRAF6 and downregulation of IL-1α and IL-1β were observed in MVA-infected immature human monocyte-derived dendritic cells (MDDCs), but not in NYVAC-infected cells [82].

NYVAC has been proposed as a candidate for a human immunodeficiency virus (HIV) vaccine. NYVAC-C ΔA52R ΔB15R ΔK7R, and NYVAC deletion mutant lacking *A52R*, *B15R* (encoding B14 protein that binds IKKβ), and *K7R* (encoding TRAF6 and IRAK2-binding protein) has been used to express HIV type 1 (HIV-1) envelope (Env) glycoprotein 120 (gp120) and GPN (Gag-Pol-Nef) clade C antigens. In mice, NYVAC-C ΔA52R ΔB15R ΔK7R induced the activation of chemokines/cytokines and migration of Nα and Nβ neutrophils to the infection site. This effect was accompanied by an increase in T cell response toward HIV antigens. The activation of virus-specific CD8^+^ T cells was triggered by Nβ neutrophils displaying an APC-like phenotype [83]. Further analyses of mice models infected with NYVAC mutants (NYVAC-C ΔA52R, NYVAC-C ΔA52R ΔK7R, or NYVAC-C ΔA52R ΔB15R) showed that the infection increased the number of CD11c^+^ major histocompatibility complex (MHCII)^+^-positive DCs in mice. Deletion of *A52R* gene influenced the migration of DCs, whereas double-gene deletion affected the migration of both DCs and neutrophils. Finally, the deletion of all *A52R*, *B15R*, and *K7R* genes not only enhanced the migration of DCs, neutrophils, and natural killer (NK) cells but also influenced chemokine release. In addition, NYVAC-C ΔA52R ΔB15R, NYVAC-C ΔA52R ΔK7R, and NYVAC-C Δ3 (triple-deletion) mutants induced CTLs. Among the double-deletion mutants, NYVAC-C ΔA52R ΔB15R not only induced a strong T CD8^+^ response but was also effective in the induction of IgG. These studies demonstrate that double or triple deletion of NF-κB inhibitors from NYVAC enhances both T cell-specific and humoral anti-HIV responses. The induction of Gag- and Pol-specific CD8^+^ T lymphocytes by NYVAC mutants showed that NYVAC-based vectors are promising anti-HIV vaccine candidates [84].

#### 5.1.3. VACV Western Reserve

Another VACV strain that can be used as a vaccine vector is VACV-WR. When modifying the VACV-WR genome, single-gene deletions are more beneficial than the deletions of multiple genes, which may decrease the immunogenicity of the vaccine [85]. One of the candidate genes that can be deleted from VACV-WR is *N1L*, encoding a Bcl-like inhibitor of NF-κB, which prevents NF-κB activation by proinflammatory cytokines including TNF-α or IL-1β [86]. Studies on intradermal murine model infection with VACV-WR devoid of *N1L* gene have shown that NF-κB is essential for CD8^+^ T cell memory and, consequently, for the efficacy of vaccines. N1 is an early VACV protein that inhibits apoptosis. Therefore, its mutation or deletion reduces the virulence of VACV and, at the same time, enhances CD8^+^ T cell response, which is desirable for antiviral protection induced by the vaccine [87]. VACV-WR vaccine can also be modified by the deletion of *K1L* NF-κB inhibitor. In mice models, K1-deficient virus induced VACV-specific CD8^+^ T cell response and prevented lethal VACV infections, despite silencing the innate immune response. Above all, at day one postinfection, the deletion mutant did not induce the expression of NF-κB-regulated genes, such as *Nfkbia* and *Tnf*. However, *Ifna4*, *Il7*, and *Nfkb2*, which are only partially controlled by NF-κB, were downregulated [88].

Recently, the importance of VACV-WR BTB-BACK-Kelch (BBK)-like protein, A55, in NF-κB modulation has been described. A55 is an NF-κB inhibitor that disturbs the p65–importin interaction and thus prevents the transcription of NF-κB-regulated genes and impairs inflammatory response. Especially, NF-κB-regulated cytokines and inflammation influence the proliferation and development of effector and memory T cells. As expected, the deletion of the *A55R* gene from VACV-WR resulted in the enhancement of CD8^+^ T cell memory, and the vaccine displayed increased immunogenicity and protected the mice challenged with VACV intranasally [89].

### 5.2. Orf Virus

ORFV, a virus belonging to the *Parapoxvirus* genus, is the causative agent of orf disease—highly contagious ecthyma. In humans, orf is an enzootic and self-limiting disease manifested as pustular dermatitis, which spontaneously resolves within 3 to 6 weeks. The ORFV infections caused in humans are frequently observed in Asia and Africa. In sheep and goats, these infections cause scabby mouth disease, which is characterized by high morbidity in infected sheep worldwide. ORFV may also infect cats, reindeers, camels, serows, and musk oxen. Mortality due to ORFV infections is rarely associated with secondary infections and aspiration pneumonia. In general, orf disease is a threat to kids and lambs and may cause farms to suffer economic losses [90,91,92,93,94]. 

The use of ORFV as a vaccine vector may constitute a novel strategy to vaccinate both permissive and nonpermissive hosts against orf, which is an alternative to the current attenuated vaccines that are inefficient or insufficiently safe. The immunomodulatory properties of ORFV, as well as its ability to replicate in various hosts, make it a good vaccine candidate. Moreover, preclinical studies have confirmed these properties of inactivated ORFV [91]. Due to the fact that ORFV does not spread systemically or neutralize antibody production, it can be successfully used as a vaccine vector for repeat immunizations [95]. The highly attenuated anticontagious ecthyma vaccine strain D1701 derived from ORFV protects sheep for 4–6 months [96,97]. When adapted to Vero cells, D1701-V can be used to deliver target genes into the *vegf-e* gene for the construction of a vaccine against pseudorabies virus (PRV), the causative agent of Aujeszky’s disease [98,99,100]. D1701-V-VP1 expressing capsid protein VP1 has been used to immunize rabbits against rabbit hemorrhagic disease virus (RHDV) [101]. Another recombinant D1701-V vector, D1701-V-RabG expressing the RABV glycoprotein, has been designed as a new antirabies vaccine for companion animals and tested on murine, dog, and cat models [97]. D1701-V-HAh5n expressing H5 hemagglutinin has also been proposed as a vaccine against avian influenza virus H5N1 and tested on mice models [102]. In addition, DNA vaccines expressing ORFV011 EEV envelope phospholipase and ORFV059 immunodominant envelope antigen F1L protein have shown enhanced immunogenicity and triggered lasting immunity in mouse models [103].

#### ORFV-IA82

Thus far, several ORFV-encoded proteins capable of inhibiting NF-κB signaling have been identified, including ORFV024 [104], ORFV002 [105,106], ORFV121 [107], ORFV073 [108], and ORFV119 [109]. Recently, ORFV020, a dsRNA-binding IFN resistance protein, displaying dsRNA adenosine deaminase activity, has been described. ORFV020 is a counterpart of VACV-WR E3, which inhibits the activation of PKR and NF-κB. Moreover, it belongs to viral IFN (VIR) resistance proteins that inhibit IFN-mediated antiviral response. Therefore, ORFV expressing ORFV020 is resistant to the activity of IFN type I and type II. Considering the conservative nature of ORFV isolates, the deletion or mutation of the *E3L* counterpart may be ideal for vaccine construction [110].

ORFV strain ORFV-IA82 is used as a vaccine against porcine epidemic diarrhea. The ORFV-PEDV-S virus expressing spike (S) proteins of porcine epidemic diarrhea virus (PEDV) was constructed using the *ORFV121* locus insertion site. This site encodes a unique parapoxviral NF-κB inhibitor, which blocks the phosphorylation of p65 and its nuclear translocation [107]. Immunization of pigs with ORFV-PEDV-S induced the production of neutralizing antibodies and PEDV-specific serum IgA and IgG. Importantly, ORFV-PEDV-S ensured protection from the clinical outcomes of the infection. Reduced virus shedding was also found upon the immunization of infected animals [111]. Furthermore, ORFV-PEDV-S vaccine has been shown to induce passive immunity in newborn piglets [112].

In addition, ORFV-IA82 can be used as an antirabies vaccine. The gene *ORFV024* encoding a unique parapoxviral inhibitor of IKK kinase and IκBα degradation [89] was used as an insertion site for the RABV glycoprotein (G) gene. Similarly, *ORFV121* gene encoding NF-κB inhibitor has been used as an insertion site for G-encoding gene. Immunization of pigs and cattle with ORFV^Δ024^RABV-G or ORFV^Δ121^RABV-G resulted in the induction of neutralizing antibodies. Of these, the ORFVΔ121 mutant was more immunogenic [113].

### 5.3. Goatpox Virus

GTPV, a member of the *Capripoxvirus* genus, is a sheep pathogen and the causative agent of the goatpox disease. Goatpox is transmitted via aerosols and insects and causes systemic infections in goats and sheep, which manifest as fever, enlargement of lymph nodes and skin, and respiratory and gastrointestinal lesions. Goatpox disease is also a source of economic loss to domestic ruminant farms. In general, GTPV is an economically important capripoxvirus in central Asia, North Africa, the Middle East, and India [114,115,116]. At present, only attenuated vaccines are available for GTPV and other capripoxviruses [117]. The capripoxvirus-based vaccines, which are obtained by serial passages, confer protective immunity for 1 year after vaccination. Capripoxviruses can also be used as vectors for vaccines against diseases caused by ruminant pathogens, such as bluetongue, Rift Valley fever, peste des petits ruminants, or rinderpest. Certain GTPV strains, such as Isiolo and Kedong, which infect goats, sheep, and cattle, are used as a universal vaccine against capripox diseases [46]. Gorgan strain-based vaccines protect cattle against lumpy skin disease. Caprivac (Jordan Bio-Industries Centre, JOVAC) is one of the vaccines used against goatpox in cattle in the Middle East [118].

#### GTPV-AV41

The existing GTPV vaccine, GTPV-AV41, contains an attenuated strain obtained by passages of GTPV-AV40 strain in the testis cells of goats and sheep. Unfortunately, it may cause generalized skin lesions and miscarriages in vaccinated animals, and thus, its use hinders distinguishing between vaccinated and infected ones [119]. Therefore, modifications of the virus are needed to improve the vaccine. It is worth noticing that the inactivation of NF-κB-related genes has been observed among capripoxvirus vaccine strains. For instance, in the GTPV Gorgan vaccine, a 1.6-kbp deletion led to the inactivation of *GTPV_144* and *GTPV_145* genes. *GTPV_144* is a counterpart of VACV-COP *A55R*, encoding Kelch repeat and BTB domain-containing protein 1, while *GTPV_145* is related to VACV-COP *B4R*, encoding ANK repeat protein [117]. Mutations in these two genes are common in capripoxviruses and are typical for vaccine strains. Since A55 inhibits CD8^+^ T cell memory, the deletion of *A55R* gene or its GTPV counterpart may improve the immunogenicity of the vaccine [89]. *GTPV_145* encodes an ANK repeat protein, a counterpart of B4 VACV-COP and EVM154 proteasome inhibitor of ectromelia virus, which interacts with Skp1 and conjugated ubiquitin and subsequently inhibits IκBα degradation. It is believed that that EVM154 may be involved in virus spread and its depletion may cause attenuation of the virus [120,121]. 

One of the insertion site candidates for GTPV-based vaccines is the 135 ORF containing an early gene, which is not essential for the viral replication in vitro and in vivo. The *135* gene encodes an 18-kDa protein, which inhibits NF-κB and apoptosis. The GTPV135 protein is a counterpart of a Bcl-like VACV-WR N1 protein. The *135* gene is a host innate immune response inhibitor and may therefore serve as an insertion site for live attenuated dual vaccines instead of the tk locus insertion site. Interestingly, the GTPV AV41 vaccine strain expressing the hemagglutinin protein of peste des petis ruminants virus (PPRV), whose gene was inserted in the ORF135 insertion site, displayed a stronger antibody neutralization response than the strain with a *tk* insertion site [122]. To improve GTPV-AV41 and prevent the side effects of the vaccine, it may be necessary to perform modifications based on the deletion of the nonessential gene. For instance, deletion of the viral *tk* gene and ORF8–18 may be beneficial for the vaccine. Among ORF8–18 homologs of VACV, the following NF-κB inhibitors can be found: ORF12, which encodes ANK repeat protein and a counterpart of B4, and ORF15, encoding a homolog of VACV IL-18BP, C12. ORF16, in turn, encodes an EGF-liked growth factor, a C11 VACV counterpart, which may activate NF-κB. In vaccinated animals, the attenuated vaccine GTPV-TK-ORF vector allowed maintaining immunogenicity and increased safety compared to wild-type GTPV-AV41. The vaccine also induced the production of neutralizing antibodies and GTPV-specific antibodies, as well as the release of IFN-γ in goats. Hence, removal of nonessential genes that are linked to apoptosis inhibition and immune modulation is considered as a factor that may improve the efficacy of the vaccine [123].

## 6. Conclusions

Generation of effective immune response and immunological memory, as well as safety, is the main concern in vaccine development. When employing virus-based vaccines, it is necessary to ensure both the complete replication cycle of the virus and proper induction of immunological memory for determining the vaccine efficiency. The loss of viral immunomodulatory proteins may affect these parameters, thus influencing efficiency. Since poxviruses modulate the activation of immune cells by affecting the NF-κB-mediated apoptosis regulation, inflammation, and immunological memory, discovering new mechanisms of NF-κB inhibition and cellular targets of poxviruses may help modify vaccine candidates to improve the efficacy of poxvirus-based vaccines and the immunological memory generated by them.

## Figures and Tables

**Table 1:** Summary of ongoing clinical trials on VACV-based vaccines against viral diseases.

Interventions	Conditions	Status	Study ID Number
Vaccination with ACAM2000	Smallpox vaccine adverse reaction	Phase 4	NCT02443623
Imvamune	MPXV infection	Phase 3	NCT02977715
MVA-NP+M1	Influenza	Phase 2	NCT03880474
Ad26.Mos.HIV MVA-mosaic gp140 DP	Healthy (HIV prevention)	Phase 1 Phase 2	NCT02315703
DNA.HTI + MVA.HTI	HIV	Phase 1	NCT03204617
ChAdOx1.HTI + MVA.HTI			
Ad26.Mos4.HIV	HIV	Phase 1	NCT03307915
MVA-mosaic			
Clade C gp140 + Mosaic gp140			
MVA.tHIVconsv3	HIV-1	Phase 1	NCT03844386
MVA.tHIVconsv4			
DNA.HTI	HIV-1	Phase 1	NCT04385875
MVA.HTI			
ChAdOx1.HTI			
IL-12 adjuvanted p24CE DNA prime	HIV/AIDS	Phase 1 Phase 2	NCT04357821
IL-12 adjuvanted DNA boost (p24CE + p55^gag^)			
MVA/HIV62B (MVA62B) boost			
single dose of two bNAbs with a TLR9 agonist			
ATI with single dose of VRC07 and 10-1074			
Raltegravir	HIV	Phase 2	NCT02336074
Vorinostat			
ChAdV63.HIVconsv			
MVA.HIVconsv			
ChAdOx1.HTI	HIV/AIDS	Phase 2	NCT04364035
MVA.HTI			
GS-9620			
Ad26.HPV16	HPV	Phase 1 Phase 2	NCT03610581
Ad26.HPV18			
MVA.HPV16/18			
ChAd155-hIi-HBV	Hepatitis B, chronic	Phase 1	NCT03866187
HBc-HBs/AS01B-4			
MVA-HBV			
ChAd3-hliNSmut	Hepatitis C	Phase 1	NCT03688061
MVA-hliNSmut			
Multi-peptide	HCT patients previously infected with CMV	Phase 2	NCT02506933
CMV-MVA vaccine			
Multi-antigen	CMV-positive	Phase 1	NCT03354728
CMV-MVA vaccine	HCT recipient	Phase 2	
Multi-peptide	Stem cell donors vaccination	Phase 2	NCT03560752
CMV-MVA vaccine			
Multi-peptide	CMV-positive	Phase 2	NCT04060277
CMV-MVA vaccine	HCT recipient		
Ad26.ZEBOV	Ebola	Phase 2	NCT02876328
MVA-BN-Filo			
rVSVΔG-ZEBOV-GP			
rVSV boost			
Ad26.ZEBOV/MVA-BN-Filo	Ebola	Phase 2	NCT04028349
Ad26.ZEBOV	Ebola	Phase 2	NCT03929757
MVA-BN-Filo			
MenACWY			
Ad26.ZEBOV vaccine	Ebola	Phase 2	NCT04186000
Ad26.ZEBOV	Hemorrhagic fever	Phase 3	NCT02661464
MVA-BN-Filo	Ebola		
Ad26.ZEBOV	Ebola	Phase 3	NCT04152486
MVA-BN-Filo			
Ad26.ZEBOV	Ebola	Phase 3	NCT04228783
MVA-BN-Filo			

Abbreviations: Ad, adenovirus; Ad26, Ad serotype 26; Ad26.HPV16, Ad26 HPV16 vaccine; Ad26.HPV18, Ad26 HPV18 vaccine; Ad26.Mos.HIV, Ad26-mosaic-HIV vaccine; Ad26.Mos4.HIV, Ad26-mosaic 4-HIV vaccine; Ad26.ZEBOV, human Ad26 expressing the Ebola virus Mayinga variant gp; AIDS, acquired immune deficiency syndrome; ATI, analytical treatment interruption; bNAbs, broadly neutralizing HIV-1 antibodies; ChAd, Chimpanzee adenovirus; ChAd155, ChAd serotype 155; ChAd155-hIi-HBV, ChAd HBV vaccine; ChAd3-hliNSmut, ChAd3 encoding NSmut linked to hli; ChAdOx1, replication-deficient ChAd vector derived from isolate Y25; ChAdOx1.HTI, ChAdOx1 expressing HTI; ChAdV63, ChAd serotype 63; ChAdV63.HIVconsv, ChAdV63 expressing HIVconsv; CMV, cytomegalovirus; DNA.HTI, plasmid DNA expressing HTI; gp, glycoprotein; gp140 DP, gp140 drug product; GS-9620, vesatolimod; HBc, hepatitis B core antigen; HBc-HBs/AS01B-4, HBV vaccine; HBs, hepatitis B surface antigen; HBV, hepatitis B virus; HCT, hematopoietic cell transplantation; HIV, human immunodeficiency virus; HIV-1, HIV type 1; HIVcons, HIV conserved antigenic regions; hli, human invariant chain; HPV, human papillomavirus; HPV16/18, human papillomavirus type 16/18; HTI, HIVACAT T cell immunogen; IL-12, interleukin-12; M1, matrix protein; MenACWY, meningococcal ACWY-tetanus toxoid conjugate vaccine; MPXV, monkeypox virus; MVA, modified vaccinia virus Ankara; MVA.HIVconsv, MVA expressing HIVconsv; MVA.HPV16/18, MVA HPV16/18 vaccine; MVA.HTI, MVA expressing HTI; MVA.tHIVconsv3, MVA.tHIVconsv4, MVA-based T-cell vaccines expressing novel HIV-1 immunogens; MVA62B, MVA component encoding HIV-1 Gag, protease, reverse transcriptase, and envelope protein gp160; MVA-BN, MVA—Bavarian Nordic; MVA-BN-Filo, MVA-BN-Filo vector; MVA-HBV, MVA HBV vaccine; MVA-hliNSmut, MVA encoding NSmut linked to hli; MVA-mosaic, MVA mosaic HIV vaccine; MVA-NP + M1, MVA encoding NP and M1; NP, nucleoprotein; NSmut, HCV nonstructural immunogen; p24CE + p55^gag^, DNA vaccines expressing p24CE and p55^gag^ immunogens; rVSVΔG-ZEBOV-GP, recombinant VSV–Zaire Ebola virus gp; TLR9, Toll-like receptor 9; VRC07, 10-1074, anti-HIV-1 bNAbs; VSV, vesicular stomatitis virus.

## Abbreviations

VARV	variola virus
VACV	vaccinia virus
ORFV	orf virus
GTPV	goatpox virus
MV	mature virus
EV	extracellular virus
ORF	open reading frame
TK	thymidine kinase
ITR	inverted terminal repetition
IFN	interferon
STING	stimulator of IFN genes
cGAS	cyclic GMP-AMP synthase
DNA-PK	DNA-dependent protein kinase
IFI16	IFN-γ-inducible protein 16
TNF	tumor necrosis factor
TNFR	TNF receptor
TRAF	TNFR-associated factor
NF-κB	nuclear factor kappa-light-chain-enhancer of activated B cells
TANK	TRAF family member-associated NF-κB activator
TBK1	TANK-binding kinase
IRF3	IFN regulatory factor 3
IκB	inhibitor κB
IKK	IκB kinase
CTL	cytotoxic T lymphocyte
NYCBH	New York City Board of Health
VACV-COP	VACV Copenhagen strain
cESC	chicken embryonic stem cell
MVA	modified VACV Ankara
MVA-BN	modified VACV Ankara - Bavarian Nordic
MPXV	monkeypox virus
CPXV	cowpox virus
CMLV	camelpox virus
BPSV	bovine papular stomatitis virus
PCPV	pseudocowpox virus
YMTV	Yaba monkey tumor virus
TPV	tanapox virus
RABV	rabies virus
V-RG	RABV glycoprotein gene
CHIKV	chikungunya virus
SCV	Sementis Copenhagen Vector
Ad	adenovirus
Ad26	Ad serotype 26
Ad26.HPV16	Ad26 HPV16 vaccine
Ad26.HPV18	Ad26 HPV18 vaccine
Ad26.Mos.HIV	Ad26-mosaic-HIV vaccine
Ad26.Mos4.HIV	Ad26-mosaic 4-HIV vaccine
Ad26.ZEBOV	human Ad26 expressing the Ebola virus Mayinga variant gp
AIDS	acquired immune deficiency syndrome
ATI	analytical treatment interruption
bNAbs	broadly neutralizing HIV-1 antibodies
ChAd	Chimpanzee adenovirus
ChAd155	ChAd serotype 155
ChAd155-hIi-HBV	ChAd HBV vaccine
ChAd3-hliNSmut	ChAd3 encoding NSmut linked to hli
ChAdOx1	replication-deficient ChAd vector derived from isolate Y25
ChAdOx1.HTI	ChAdOx1 expressing HTI
ChAdV63	ChAd serotype 63
ChAdV63.HIVconsv	ChAdV63 expressing HIVconsv
CMV	cytomegalovirus
DNA.HTI	plasmid DNA expressing HTI
gp	glycoprotein
gp140 DP	gp140 drug product
GS-9620	vesatolimod
HBc	hepatitis B core antigen
HBc-HBs/AS01B-4	HBV vaccine
HBs	hepatitis B surface antigen
HBV	hepatitis B virus
HCT	hematopoietic cell transplantation
HIV	human immunodeficiency virus
HIV-1	HIV type 1
HIVcons	HIV conserved antigenic regions
hli	human invariant chain
HPV	human papillomavirus
HPV16/18	human papillomavirus type 16/18
HTI	HIVACAT T cell immunogen
IL-12	interleukin-12
M1	matrix protein
MenACWY	meningococcal ACWY-tetanus toxoid conjugate vaccine
MVA.HIVconsv	MVA expressing HIVconsv
MVA.HPV16/18	MVA HPV16/18 vaccine
MVA.HTI	MVA expressing HTI
MVA.tHIVconsv3	MVA-based T-cell vaccine expressing novel HIV-1 immunogens
MVA.tHIVconsv4	MVA-based T-cell vaccine expressing novel HIV-1 immunogens
MVA62B	MVA component—encoding HIV-1 Gag, protease, reverse transcriptase, and envelope gp160
MVA-BN-Filo	MVA-BN-Filo vector
MVA-HBV	MVA HBV vaccine
MVA-hliNSmut	MVA encoding NSmut linked to hli
MVA-mosaic	MVA mosaic HIV vaccine
MVA-NP + M1	MVA encoding NP and M1
NP	nucleoprotein
NSmut	HCV nonstructural immunogen
p24CE + p55^gag^	DNA vaccines expressing p24CE and p55^gag^ immunogens
rVSVΔG-ZEBOV-GP	recombinant VSV–Zaire Ebola virus gp
TLR9	Toll-like receptor 9
VRC07, 10-1074	anti-HIV-1 bNAbs
VSV	vesicular stomatitis virus
NLS	nuclear localization sequence
IL-1β	interleukin-1β
IL-18	interleukin-18
TNF-α	tumor necrosis factor-α
PRR	pattern recognition receptor
RIG-I	retinoic acid-inducible gene-I
TGF-β	transforming growth factor-β
TAK1	TGF-β-activated kinase 1
Skp1	S-phase kinase-associated protein 1
SCF	Skp1-Cul1-F-box
β-TrCP	β-transducin repeat-containing protein
NIK	NF-κB-inducing kinase
Ac	acetyl group
CBP	CREB-binding protein
CpG	cytosine–guanine dinucleotide
ERK2	extracellular signal-regulated kinase 2
IKKα	IκB kinase α
IKKβ	IκB kinase β
IKKγ	IκB kinase γ
IL-18R	IL-18 receptor
IL-1βR	IL-1β receptor
IRAK1	IL-1R-associated kinase 1
IRAK2	IL-1R-associated kinase 2
LPS	lipopolysaccharide
Mal	MyD88-adapter-like
MyD88	myeloid differentiation primary response gene 88
P	phosphate group
Pol III	polymerase III
TLR3	Toll-like receptor 3
TLR4	Toll-like receptor 4
TLR7	Toll-like receptor 7
TLR8	Toll-like receptor 8
TRAM	TRIF-related adapter molecule
TRIF	Toll-IL-1R-domain-containing adapter-inducing interferon-β
Ub	Ub-ubiquitin moieties
VACV-WR	VACV Western Reserve strain
β-TrCP	β-transducin repeat-containing protein
CVA	chorioallantois VACV Ankara strain
IL-1BP	IL-1 binding protein
Bcl-2	B-cell lymphoma 2
VGF	VACV growth factor
EGFR	epidermal growth factor receptor
HEK 293T	human embryonic kidney 293 cells transformed with large T antigen
CHO	Chinese hamster ovary cells
RK13	rabbit kidney 13 cells
CPXV-BR	CPXV Brighton Red strain
ANK	ankyrin repeat
MYXV	myxoma virus
MEFs	mouse embryonic fibroblasts
PKR	protein kinase R
MEK	mitogen-activated protein kinase kinase
APC	antigen presenting cell
ATF3	activating transcription factor 3
MDDC	monocyte-derived dendritic cell
gp120	glycoprotein 120
GPN	Gag-Pol-Nef
MHCII	major histocompatibility complex class II
NK	natural killer cell
BBK	BTB-BACK-Kelch
PRV	pseudorabies virus
RHDV	rabbit hemorrhagic disease virus
D1701-V-RabG	recombinant D1701 ORFV strain expressing RABV glycoprotein
D1701-V-HAh5n	recombinant D1701 ORFV strain expressing H5 hemagglutinin
VIR	viral IFN resistance
PEDV	porcine epidemic diarrhea virus
S	spike protein
ORFV-PEDV-S	
ORFVΔ024RABV-G	ORFV Δ024 mutant expressing RABV glycoprotein
ORFVΔ121RABV-G	ORFV Δ121 mutant expressing RABV glycoprotein
PPRV	peste des petis ruminants virus
